# Supplementary material for: Resolving the full spectrum of human genome variation using Linked-Reads
Source: Genome Res. 2019 Apr;29(4):635–45. doi: 10.1101/gr.234443.118 (PMC6442396; doi:10.1101/gr.234443.118)
Supplement: Supplemental Material [file supp_gr.234443.118_Supplemental_longranger-2.2.zip › longranger-2.2.tar.bz2!/lr2.2-public/lib/stan/cmdstan/stan/doc/api/html/head_8hpp_source.html]

Stan: src/stan/math/matrix/head.hpp Source File


|  |  |
| --- | --- |
|  | Stan  2.5.0  probability, sampling & optimization |


- Main Page
- Related Pages
- Namespaces
- Classes
- Files

- File List
- File Members

All Classes Namespaces Files Functions Variables Typedefs Enumerations Enumerator Friends Macros Pages

- src
- stan
- math
- matrix

head.hpp

Go to the documentation of this file.

1 #ifndef STAN\_\_MATH\_\_MATRIX\_\_HEAD\_HPP

2 #define STAN\_\_MATH\_\_MATRIX\_\_HEAD\_HPP

3

4 #include <stan/math/matrix/Eigen.hpp>

5 #include <vector>

6 #include <stan/math/error\_handling/matrix/check\_column\_index.hpp>

7 #include <stan/math/error\_handling/matrix/check\_row\_index.hpp>

8 #include <stan/math/error\_handling/matrix/check\_std\_vector\_index.hpp>

9

10 namespace stan {

11  namespace math {

12

21  template <typename T>

22  inline

23  Eigen::Matrix<T,Eigen::Dynamic,1>

24  head(const Eigen::Matrix<T,Eigen::Dynamic,1>& v,

25  size\_t n) {

26  if (n != 0)

27  stan::math::check\_row\_index("head(%1%)",n,v,"n",(double\*)0);

28  return v.head(n);

29  }

30

39  template <typename T>

40  inline

41  Eigen::Matrix<T,1,Eigen::Dynamic>

42  head(const Eigen::Matrix<T,1,Eigen::Dynamic>& rv,

43  size\_t n) {

44  if (n != 0)

45  stan::math::check\_column\_index("head(%1%)",n,rv,"n",(double\*)0);

46  return rv.head(n);

47  }

48

57  template <typename T>

58  std::vector<T> head(const std::vector<T>& sv,

59  size\_t n) {

60  if (n != 0)

61  stan::math::check\_std\_vector\_index("head(%1%)",n,sv,"n",(double\*)0);

62

63  std::vector<T> s;

64  for (size\_t i = 0; i < n; ++i)

65  s.push\_back(sv[i]);

66  return s;

67  }

68

69

70  }

71 }

72

73 #endif

stan::math::check\_std\_vector\_index

bool check\_std\_vector\_index(const char \*function, size\_t i, const std::vector< T\_y > &y, const char \*name, T\_result \*result)

Return true if the specified index is valid in std vector.

**Definition:** check\_std\_vector\_index.hpp:25

check\_column\_index.hpp

stan::math::check\_column\_index

bool check\_column\_index(const char \*function, size\_t i, const Eigen::Matrix< T\_y, R, C > &y, const char \*name, T\_result \*result)

Return true if the specified index is a valid column of the matrix.

**Definition:** check\_column\_index.hpp:25

check\_std\_vector\_index.hpp

stan::math::head

Eigen::Matrix< T, Eigen::Dynamic, 1 > head(const Eigen::Matrix< T, Eigen::Dynamic, 1 > &v, size\_t n)

Return the specified number of elements as a vector from the front of the specified vector...

**Definition:** head.hpp:24

check\_row\_index.hpp

stan::math::check\_row\_index

bool check\_row\_index(const char \*function, size\_t i, const Eigen::Matrix< T\_y, R, C > &y, const char \*name, T\_result \*result)

Return true if the specified index is a valid row of the matrix.

**Definition:** check\_row\_index.hpp:25

Eigen.hpp


---

[ Stan Home Page ]

*© 2011–2014,
Stan Development Team.*
